# PD-L1 expression predicts the efficacy of PD-1 blockade plus chemotherapy versus chemotherapy alone in treatment-naïve advanced or metastatic gastric cancer: a pooled analysis of reconstructed individual patient-level data from two randomized trials

**DOI:** 10.3389/fcell.2025.1636288

**Published:** 2025-07-03

**Authors:** Wei Zhou, Zeng-Zhi Cai, Zhuolin Fan, Xu Zheng, Yu-Tong Chen

**Affiliations:** ^1^ State Key Laboratory of Oncology in South China, Department of Medical Oncology, Collaborative Innovation Center for Cancer Medicine, Research Unit of Precision Diagnosis and Treatment for Gastrointestinal Cancer, Sun Yat-sen University Cancer Center, Chinese Academy of Medical Sciences, Guangzhou, China; ^2^ Department of General Surgery, The Fourth Affiliated Hospital of China Medical University, Shenyang, China; ^3^ Shenyang Kingmed Diagnostics Co., Ltd., Shenyang, China; ^4^ Faculty of Medical Science, Jinan University, Guangzhou, China

**Keywords:** advanced gastric cancer, programmed cell death Protein-1 blockade, chemotherapy, programmed cell death-ligand 1 expression, combined positive score, randomized controlled trials

## Abstract

**Background:**

Chemotherapy alone exhibits suboptimal efficacy in patients with treatment-naïve advanced gastric cancer (GC). Randomized controlled trials (RCTs) have demonstrated that combining Programmed Cell Death Protein-1 (PD-1) blockade with chemotherapy significantly improves overall survival (OS) compared to chemotherapy alone. However, the efficacy of PD-1 inhibitors in patients with low Programmed Cell Death-Ligand 1 (PD-L1) expression remains unclear.

**Methods:**

Electronic databases were searched for RCTs comparing PD-1/PD-L1 inhibitors plus chemotherapy to placebo plus chemotherapy or chemotherapy alone in treatment-naïve advanced gastric or gastroesophageal junction adenocarcinoma patients. Individual patient-level data (IPD) for overall survival (OS) and progression-free survival (PFS) were reconstructed. The KMSubtraction algorithm was employed to derive IPD for the PD-L1-low subgroup. Treatment effects in PD-L1-high and PD-L1-low subgroups were evaluated using Cox proportional hazards models with shared frailty to account for between-study heterogeneity. Interaction tests were performed to assess differences in treatment effects between these subgroups.

**Results:**

Nine RCTs were included in the qualitative analysis. A combined positive score (CPS) of 5 was selected as the cutoff for analysis, with CheckMate 649 and ORIENT-16 trials included. In the CPS<5 subgroup, OS (CheckMate 649: HR = 0.97, 95% CI 0.81–1.17, P = 0.758; ORIENT-16: HR = 0.94, 95% CI 0.68–1.31, P = 0.725) and PFS (CheckMate 649: HR = 0.95, 95% CI 0.79–1.14, P = 0.580; ORIENT-16: HR = 0.73, 95% CI 0.52–1.01, P = 0.055) did not significantly differ between patients receiving PD-1 blockade plus chemotherapy and those receiving chemotherapy alone. Pooled analysis of reconstructed OS IPD from CheckMate 649 and ORIENT-16 (N = 2,231) revealed that PD-1 blockade significantly improved OS in the CPS≥5 subgroup (HR = 0.69, 95% CI 0.60–0.79, P < 0.001), but not in the CPS<5 subgroup (HR = 0.96, 95% CI 0.82–1.13, P = 0.643). Interaction tests showed a significantly attenuated treatment effect on OS in the CPS<5 subgroup compared to the CPS≥5 subgroup (Pinteraction = 0.002). Similar findings were observed in the pooled analysis of PFS data (Pinteraction = 0.011).

**Conclusion:**

The addition of PD-1 inhibitors to first-line chemotherapy provides minimal benefit in patients with CPS<5. Therefore, PD-1 inhibitors should be individualized for this patient subset.

## Introduction

Gastric cancer (GC) is the fifth most common cancer worldwide, with an estimated 1.1 million new cases and 769,000 deaths in 2020 (Sung et al., 2021). For decades, fluoropyrimidine and platinum-based doublet chemotherapy has been the most frequently used first-line treatment for patients with HER-2-negative advanced GC (Alsina et al., 2023). During this timeframe, the median overall survival (OS) was unsatisfactory, with a median OS less than 12 months (Wagner et al., 2017). Recently, the advent of immune checkpoint inhibitors has significantly transformed the treatment for advanced GC (Zhang et al., 2022; Wang et al., 2023). The ATTRACTION-02 study found that the Programmed Cell Death Protein-1 (PD-1) inhibitor nivolumab resulted in prolonged OS in patients with advanced GC refractory to standard treatment (Kang et al., 2017). In the first-line setting, the CheckMate 649 study has demonstrated that the addition of chemotherapy significantly improved OS and progression-free survival (PFS) in patients with treatment-naïve advanced GC, leading to the US Food and Drug Administration (FDA)’s approval of nivolumab plus chemotherapy for the first-line treatment of advanced GC (Janjigian et al., 2021; FDA, 2025). Additionally, several randomized clinical trials (RCTs) have shown the superior efficacy of anti-PD-1 therapy plus chemotherapy versus chemotherapy alone in patients with treatment-naïve advanced GC (Kang et al., 2022; Moehler et al., 2023; Rha et al., 2023; Xu et al., 2021).

Despite the partial success of anti-PD-1–chemotherapy combinations in first-line advanced GC, 40%–50% of patients still do not respond and median OS improvements remain modest (about 2–3 months), highlighting the urgent need for effective biomarkers to guide patient selection, with PD-L1 CPS being more predictive than TPS. (Janjigian et al., 2021; Kang et al., 2022; Rha et al., 2023; Havel et al., 2019; Wang and Xu, 2023; Martin and Märkl, 2019; Kulangara et al., 2019). Although the CheckMate 649 trial revealed that patients with high PD-L1 expression (CPS≥5) benefitted better from the addition of nivolumab to chemotherapy than those with low PD-L1 expression (CPS<5) (FDA, 2025). However, the FDA’s approval of nivolumab plus chemotherapy for first-line advanced gastric cancer did not mandate PD-L1 testing (FDA, 2025), meaning many patients with low PD-L1 expression (CPS<5) may receive a treatment not specifically suited for them. Given the strong correlation between PD-1 blockade efficacy and PD-L1 expression levels shown in CheckMate 649 and other studies, identifying patients most likely to benefit from this combination therapy is crucial. Moreover, most clinical trials have not reported detailed outcomes or Kaplan-Meier (KM) survival curves for the PD-L1-low subgroup. This data gap makes it difficult to draw clear conclusions about the potential benefits or lack thereof for patients with low PD-L1 expression. Without reliable evidence for this subgroup, clinicians face significant uncertainties in decision-making, especially regarding whether to use PD-1 blockade plus chemotherapy for patients with CPS<5. This missing data also hampers the development of more personalized treatment strategies and may lead to unnecessary treatment-related toxicities and costs for patients unlikely to benefit.

**FIGURE 1:**
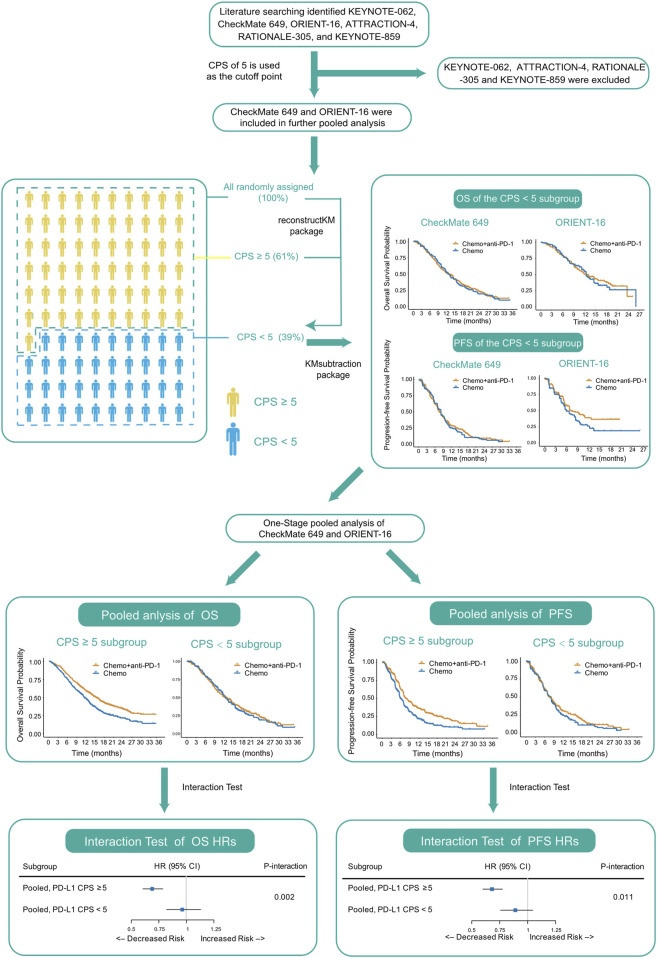
Pipeline of analyses performed in this study. Abbreviations: CPS, Combined Positive Score; OS, overall survival; PFS, progression-free survival; HR, hazard ratio.

To address this issue, we first searched for and collected published RCTs studying PD-1 blockade plus chemotherapy versus chemotherapy alone in treatment-naïve advanced GC, reconstructed individual patient-level data (IPD) from the previously reported KM curves of the all-randomized population and the PD-L1-high subgroup, and further derived the IPD of the PD-L1-low subgroup based on the reconstructed IPD. We then performed a pooled analysis of these data and assessed whether the treatment effect of PD-1 blockade plus chemotherapy versus chemotherapy alone differed between PD-L1-high and PD-L1-low subgroups. The pipeline of analyses performed in this study is shown in Figure 1.

## Methods

### Study selection

The PubMed database was searched for RCTs published between January 1, 2000, and March 1, 2023, using the following search string: ((((first-line) OR (previously untreated)) AND (nivolumab OR pembrolizumab OR toripalimab OR camrelizumab OR tislelizumab OR sintilimab OR serplulimab OR cemiplimab OR atezolizumab OR avelumab OR durvalumab OR (PD-1) OR (PD-L1))) AND (((gastric) OR (gastroesophageal junction)) OR (gastroesophageal junction))) AND ((“2000/01/01”[Date - Publication]: “2023/03/01”[Date - Publication]))). The American Society of Clinical Oncology and European Society for Medical Oncology Congress websites were also searched for publications to identify results from clinical trials that have not yet been published in peer-reviewed journals using the same search string. RCTs investigating the efficacy of PD-1/PD-L1 inhibitors plus chemotherapy versus placebo plus chemotherapy or chemotherapy alone in patients with treatment-naïve advanced gastric or gastro-esophageal junction adenocarcinoma were included. Retrospective studies, single-arm phase I and II trials, and neoadjuvant or adjuvant setting trials were excluded.

### Reconstruction of IPD

DigitizeIt software version 2.2 (http://www.digitizeit.de/) was used to scan published OS and PFS KM plots from eligible trials. Risk tables and event numbers were manually curated. Subsequently, to solve the inverted KM equations, these data were input into an algorithm based on iterative numerical methods, as implemented in the R package reconstructKM (Guyot et al., 2012).

For trials lacking KM curves for the population with low PD-L1 expression, the R package KMSubtraction was used to retrieve survival data. KMSubtraction is a workflow used to derive unreported subgroup survival data from known subgroups (Zhao et al., 2022a). For this study, it was used to derive data for subgroups with low PD-L1 expression from the data of all-comers and subgroups with high PD-L1 expression. Minimal-cost bipartite matching was used as the primary matching algorithm.

### Quality assessment of data reconstruction and matching

The quality of reconstruction was evaluated before performing a pooled analysis. Reconstructed KM curves for all-comers and subgroups with high PD-L1 expression were compared with the original published KM curves. KM curves were evaluated according to OS and PFS hazard ratios (HRs) and median OS and PFS. The accuracy of the reconstructed data was further validated by comparing KMSubtraction-derived KM curves and HRs for subgroups with low PD-L1 expression to the original published HRs. Empirical cumulative distribution plots and Bland-Altman plots were used to demonstrate discrepancies in the follow-up time between matched pairs to evaluate the effectiveness of matching (Zhao et al., 2022a). The KM curves of the matched cohorts were also plotted. The limits of error for KMSubtraction were determined by conducting Monte Carlo simulations with 10,000 iterations.

### Pooled analysis of OS and PFS

One-stage pooled analyses using reconstructed and derived IPD were conducted to elucidate whether the treatment effect of PD-1 blockade plus chemotherapy versus chemotherapy alone differed between the PD-L1-high and PD-L1-low subgroups. In all analyses, the primary outcome was prespecified as OS and the secondary outcome was PFS. Between-study heterogeneity was accounted by incorporating a random-effects term using the shared-frailty model. The gamma-distributed frailty was also used. HRs and the corresponding 95% confidence intervals (CIs) were computed using a Cox proportional hazards regression model.

All analyses were conducted in R, version 4.1.0, using the survival, ggplot2, survminer, reconstructKM, KMSubtraction, and frailtyEM packages. Statistical significance was set at a two-sided P < 0.05.

## Results

### Overview of included trials

Six RCTs met the selection criteria and were included in the analysis: KEYNOTE-062 (Shitara et al., 2020), CheckMate 649 (Janjigian et al., 2021), ORIENT-16 (Xu et al., 2021), Attraction-4 (Kang et al., 2022), RATIONALE-305 (Moehler et al., 2023), and KEYNOTE-859 (Rha et al., 2023) (Supplementary Figure S1; Table 1). Dako 22C3 was the most commonly used PD-L1 IHC assay (n = 3 [KEYNOTE-062, KEYNOTE-859, and ORIENT-16]), followed by the 28-8 (n = 2 [CheckMate 649 and ATTRACTION-4]) and SP263 (n = 1 [RATIONALE-305]) assays. Except for KEYNOTE-062, the other five trials achieved positive results that PD-1 blockade plus chemotherapy showed superior efficacy to chemotherapy alone for patients with treatment-naïve advanced GC. Among them, CheckMate 649 reported OS and PFS KM curves for the all-randomized population and the CPS≥1 and CPS≥5 subgroups, but not for the CPS<1 or CPS<5 subgroups; however, it reported OS HRs for the CPS<1, CPS<5, and CPS<10 subgroups, and the results showed no significant benefits of PD-1 blockade plus chemotherapy in terms of OS in all three subgroups. ORIENT-16 reported OS and PFS KM curves for the all-randomized population and the CPS≥5 subgroup, but not for the CPS<5 subgroup. This trial also did not report OS or PFS HRs for the CPS<5 subgroup. ATTRACTION-4 only reported OS and PFS KM curves for the all-randomized population; OS and PFS HRs were reported for the TPS≥1%, and <1% subgroups, but the treatment effect did not differ between these subgroups. RATIONALE-305 released only OS KM curves and OS HRs for the SP263 assay-based PD-L1-positive subgroup. KEYNOTE-859 only reported OS and PFS KM curves for the all-randomized population and also reported OS HRs for the CPS<1 and CPS<10 subgroups, both of which showed no significant benefits from PD-1 blockade plus chemotherapy. Ultimately, a CPS of 5 was determined to be the sole cutoff with sufficient KM curves to allow pooled analysis, and CheckMate 649 and ORIENT-16 were finally included.

**TABLE 1:** Summary of the published randomized trials included in the analysis.

Trial	Population	Treatment	*N*	PD-L1 assay	Reported outcomes (as KM plots)
CheckMate 649	Global (Asian, 22.5%)	Nivolumab + FOLFOX/XELOX	789	IHC 28–8	OS (1) All patients (2) PD-L1 CPS ≥1 (3) PD-L1 CPS ≥5 PFS (1) All patients (2) PD-L1 CPS ≥1 (3) PD-L1 CPS ≥5
		FOLFOX/XELOX	792		
ORIENT-16	Chinese	Sintilimab + XELOX	327	IHC 22C3	OS (1) All patients (2) PD-L1 CPS ≥5 PFS (1) All patients (2) PD-L1 CPS ≥5
		Placebo + XELOX	323		
KEYNOTE-062	Global (Asian, 24.7%)	Pembrolizumab + PF	257	IHC 22C3	OS (1) PD-L1 CPS ≥1 (2) PD-L1 CPS ≥10 PFS (1) PD-L1 CPS ≥1 (2) PD-L1 CPS ≥10
		Placebo + PF	250		
ATTRACTION-4	Asian	Nivolumab + SOX/XELOX	362	IHC 28–8	OS: All patients PFS: All patients
		Placebo + SOX/XELOX	362		
RATIONALE 305	Global (Asian, 73.8%)	Tislelizumab + PF/XELOX	274	IHC SP263	OS: PD-L1 vCPS ≥5 PFS: PD-L1 vCPS ≥5
		Placebo + PF/XELOX	272		
KEYNOTE-859	Global (Asian, 33.2%)	Pembrolizumab + PF/XELOX	790	IHC 22C3	OS: All patients PFS: All patients
		Placebo + PF/XELOX	789		

Abbreviations: CPS, combined positive score; HR, hazard ratio; IHC, immunohistochemistry; KM, Kaplan-Meier; OS, overall survival; PD-1, Programmed Cell Death-Protein 1; PD-L1, Programmed Cell Death-Ligand 1; PFS, progression-free survival; vCPS, SP263 assay-based PD-L1; FOLFOX, 5-fluorouracil/leucovorin/oxaliplatin; XELOX, capecitabine/oxaliplatin; SOX, S-1/oxaliplatin.

### Quality assessment of data reconstruction and matching

The reconstructed IPD resulted in OS and PFS HRs and median OS and PFS that were similar to those of the originally reported curves of the all-randomized population and the CPS≥5 subgroup in both CheckMate 649 and ORIENT-16 (Supplementary Table S1 **)**. Matched pairs on empirical cumulative distributions and Bland-Altman plots (with means of absolute differences in follow-up time approximating 0) had few discrepancies for the derived IPD of the CPS<5 subgroup. Furthermore, a near-complete overlap (with HRs and log-rank tests approximating 1) was observed in the KM plots between the matched cohorts (Supplementary Figures S2–5). For each implementation of KMSubtraction, small and negligible limits of error between the reconstructed unmatched plots and original unreported plots were demonstrated via simulations from 10,000 Monte Carlo iterations (mean |ln(HR)| = 0.017 for both treatment arms in CheckMate 649; mean |ln(HR)| = 0.020 and 0.021 for the PD-1 blockade plus chemotherapy and chemotherapy alone arms, respectively, in ORIENT-16).

### OS in the CPS<5 subgroup in CheckMate 649 and ORIENT-16

**FIGURE 2:**
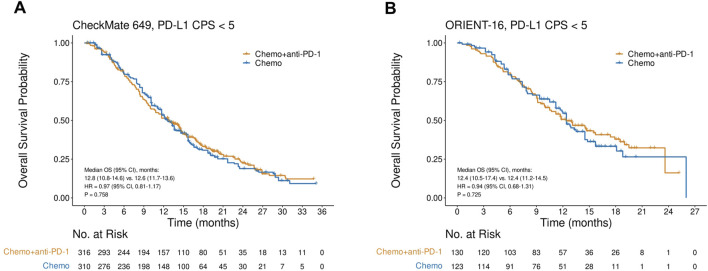
Kaplan-Meier plots for overall survival in the PD-L1 CPS<5 subgroups in CheckMate 649 **(A)** and ORIENT-16 **(B)** based on reconstructed individual patient-level data. Abbreviations: CPS, Combined Positive Score; OS, overall survival; HR, hazard ratio; Chemo, chemotherapy; Chemo + anti-PD-1, chemotherapy plus PD-1 blockade.

In the CheckMate 649 study in the CPS<5 subgroup, there was no significant difference in OS between patients treated with nivolumab plus chemotherapy and those treated with chemotherapy alone (HR = 0.97, 95% CI, 0.81–1.17, P = 0.758), with a median OS of 12.8 (95% CI, 10.8–14.6) versus 12.6 (95% CI, 11.7–13.6) months, respectively (Figure 2A). Similarly, in the ORIENT-16 study in the subgroup of CPS<5, the OS did not improve significantly in patients treated with the PD-1 inhibitor sintilimab in combination with chemotherapy compared to those treated with chemotherapy alone (HR = 0.94, 95% CI, 0.68–1.31, P = 0.725), with a median OS of 12.4 (95% CI, 10.5–17.4) versus 12.4 (95% CI, 11.2–14.5) months, respectively (Figure 2B).

### PFS in the CPS<5 subgroup in CheckMate 649 and ORIENT-16

**FIGURE 3:**
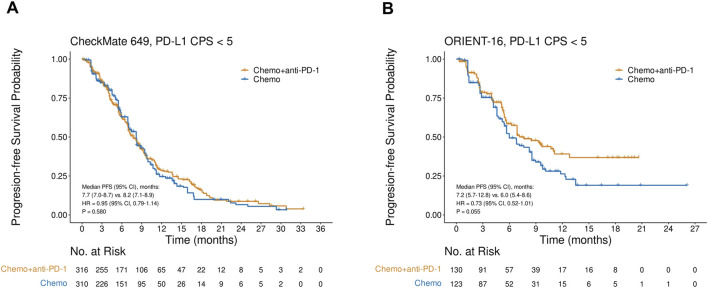
Kaplan-Meier plots for progression-free survival in the PD-L1 CPS<5 subgroups in **(A)** CheckMate 649 and **(B)** ORIENT-16 based on reconstructed individual patient-level data. Abbreviations: CPS, Combined Positive Score; PFS, progression-free survival; HR, hazard ratio; Chemo, chemotherapy; Chemo + anti-PD-1, chemotherapy plus PD-1 blockade.

In CheckMate 649, the CPS<5 subgroup gained no significant PFS improvements with the addition of nivolumab to chemotherapy (HR = 0.95, 95% CI, 0.79–1.14, P = 0.580), and the median PFS in the nivolumab plus chemotherapy arm and chemotherapy-only arm were 7.7 (95% CI, 7.0–8.7) and 8.2 (95% CI, 7.1–8.9) months, respectively (Figure 3A). Similarly, in the CPS<5 subgroup in the ORIENT-16 study, there was no significant difference in PFS between patients treated with sintilimab plus chemotherapy and those treated with chemotherapy alone (HR = 0.73, 95% CI, 0.52–1.01, P = 0.055). The median PFS was 7.2 (95% CI, 5.7–12.8) months in the sintilimab plus chemotherapy arm and 6.0 (95% CI, 5.4–8.6) months in the chemotherapy-only arm (Figure 3B).

### Pooled analysis of CheckMate 649 and ORIENT-16 based on PD-L1 status

**FIGURE 4:**
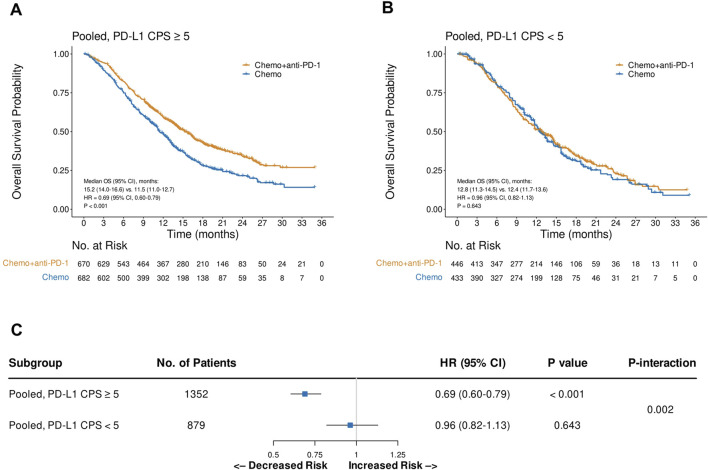
Kaplan-Meier plots for overall survival in the one-stage pooled analysis of CheckMate 649 and ORIENT-16 in the PD-L1 CPS≥5 **(A)** and CPS<5 **(B)** subgroups, and the interaction test results regarding the difference in treatment effects between the CPS≥5 and CPS<5 subgroups **(C)**. Abbreviations: CPS, Combined Positive Score; OS, overall survival; HR, hazard ratio; Chemo, chemotherapy; Chemo + anti-PD-1, chemotherapy plus PD-1 blockade.

**FIGURE 5:**
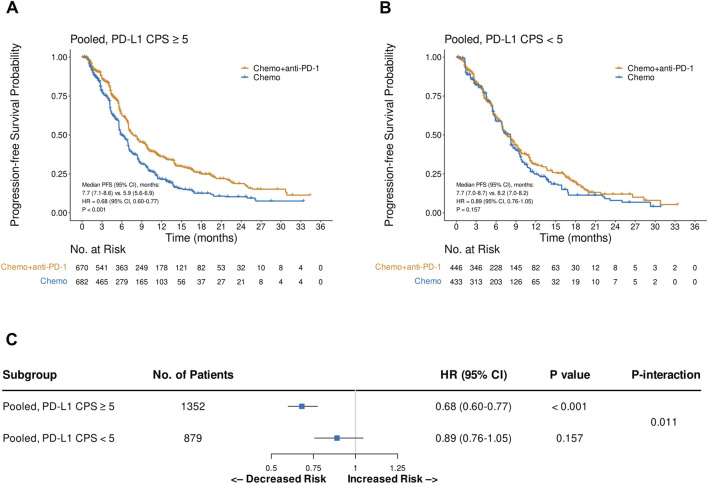
Kaplan-Meier plots for progression-free survival in the one-stage pooled analysis of CheckMate 649 and ORIENT-16 in the PD-L1 CPS≥5 **(A)** and CPS<5 **(B)** subgroups, and the interaction test results regarding the difference in treatment effects between the CPS≥5 and CPS<5 subgroups **(C)**. Abbreviations: CPS, Combined Positive Score; PFS, progression-free survival; HR, hazard ratio; Chemo, chemotherapy; Chemo + anti-PD-1, chemotherapy plus PD-1 blockade.

Pooled analysis of the reconstructed OS IPD from CheckMate 649 and ORIENT-16 showed that the addition of PD-1 blockade to chemotherapy significantly improved patient OS in the CPS≥5 subgroup (HR = 0.69, 95% CI, 0.60–0.79, P < 0.001), with a median OS of 15.2 (95% CI, 14.0–16.6) versus 11.5 (95% CI, 11.0–12.7) months, respectively (Figure 4A). In contrast, in the pooled CPS<5 subgroup, there was no significant difference in OS between patients treated with PD-1 blockade plus chemotherapy and those treated with chemotherapy alone (HR = 0.96, 95% CI, 0.82–1.13, P = 0.643, Figure 4B). The interaction test revealed that the treatment effect on OS significantly attenuated in the CPS<5 subgroup than in the CPS≥5 subgroup (P_interaction_ = 0.002, Figure 4C). Similarly, in the pooled analysis of the reconstructed PFS IPD from CheckMate 649 and ORIENT-16, the addition of PD-1 blockade to chemotherapy significantly improved patient OS in the CPS≥5 subgroup (HR = 0.68, 95% CI, 0.60–0.77, P < 0.001, Figure 5A), but not in the CPS<5 subgroup (HR = 0.89, 95% CI, 0.76–1.05, P = 0.157, Figure 5B). The interaction test showed that the treatment effect on PFS was significantly less prominent in the CPS<5 subgroup than in the CPS≥5 subgroup (P_interaction_ = 0.011, Figure 5C).

## Discussion

Recently, multiple RCTs have shown that PD-1 blockade plus chemotherapy outperforms chemotherapy alone as a first-line therapy for patients with advanced GC, especially for those with high PD-L1 expression levels (Janjigian et al., 2021; Kang et al., 2022; Moehler et al., 2023; Rha et al., 2023; Xu et al., 2021; Shitara et al., 2020). However, most RCTs did not report survival data or KM curves for patients with low PD-L1 expression; therefore, it remains unclear whether the treatment effect in the all-randomized population is largely driven by that in the CPS≥5 subgroup (Yoon et al., 2022). Therefore, we reconstructed the IPD of the CPS≥5 and CPS<5 subgroups from two large-scale RCTs: CheckMate 649 and ORIENT-16. The reconstructed data revealed that, compared to the CPS≥5 subgroup, the treatment effect in the CPS<5 subgroup was significantly less pronounced in terms of overall survival (OS) and progression-free survival (PFS).

These findings provide robust evidence for the utility of the PD-L1 CPS as a biomarker for predicting the efficacy of PD-1 blockade plus chemotherapy in patients with treatment-naïve advanced GC. Therefore, it is recommended that the PD-L1 CPS be routinely tested prior to first-line therapy for patients with advanced GC. For patients with CPS≥5, PD-1 blockade in combination with chemotherapy should be considered the standard first-line treatment. In contrast, for those with a CPS<5, the lack of efficacy of adding PD-1 blockade to chemotherapy should be part of the informed discussion of treatment options with patients, Decisions regarding the use of anti-PD-1 therapy should be made on a case-by-case basis, taking into account each patient’s specific circumstances, such as socioeconomic status, alternative therapeutic options, and predisposition to immune-related adverse events.

In the subgroup analyses of CheckMate 649 and KEYNOTE-859, PD-1 blockade plus chemotherapy did not improve OS compared to chemotherapy alone in patients with CPS<1 (HR = 0.95 and 0.92, respectively) (Janjigian et al., 2021; Rha et al., 2023). Therefore, determining whether patients with CPS 1–4 still benefit from PD-1 blockade plus chemotherapy is of interest. In a recent study, Zhao et al. reconstructed IPD from CheckMate 649 and found that the addition of nivolumab to chemotherapy did not improve OS and PFS in patients with CPS 1–4 (HR = 0.95 and 0.96, respectively) (Zhao et al., 2022b), further suggesting that neither the CPS<1 nor CPS 1–4 subgroups benefit from this combination treatment. Subgroup analysis of CheckMate 649 showed that high microsatellite instability and high tumor mutational burdens were associated with greater survival benefits from PD-1 blockade plus chemotherapy versus chemotherapy alone (Janjigian et al., 2021; Lei et al., 2022); and whether the incorporation of these features with PD-L1 expression can further improve patient selection is worthy of further investigation (Chen et al., 2023). These results have important implications for clinical practice, suggesting that for patients with CPS<5, PD-1 blockade plus chemotherapy may not be the optimal choice. Clinical decisions should take into account individual patient characteristics, such as certain tumor biology features, including microsatellite instability status and tumor mutational burden levels, as well as features of the immune microenvironment, to more precisely select appropriate treatment strategies.

Our findings highlight the urgent need to need to develop efficacious therapeutics to improve the survival of patients with PD-L1-low advanced GC. Some combination regimens have shown promising preliminary results. For instance, a recent phase 2 study investigated the efficacy of regorafenib, a potent inhibitor of angiogenic and oncogenic kinases, in combination with the PD-1 inhibitor camrelizumab and chemotherapy in patients with treatment-naïve advanced GC, which revealed a favorable objective response rate of 61.5% in the CPS<1 subgroup (Peng et al., 2021). The upregulation of DKK1 impairs CD8^+^ T cell functions and promotes MDSC-mediated immunosuppression (Shi et al., 2022). In a recent phase 2 study, the combination of a DKK1 inhibitor with the PD-1 inhibitor tislelizumab and first-line chemotherapy yielded a remarkable objective response rate of 100% in patients with DKK1-high and PD-L1-low GC (Klempner et al., 2022). These regimens warrant further confirmatory investigation, and future research should explore the role of these biomarkers in patient selection and develop more personalized treatment options for patients with PD-L1-low expression.

This study has some limitations. First, we analyzed the reconstructed IPD rather than the original IPD. However, the method used for IPD reconstruction has been validated in previous studies with excellent accuracy and reproducibility (Wang et al., 2021). Second, PD-L1 CPS was not a prespecified stratification factor in either CheckMate 649 or ORIENT-16; therefore, there may be confounding factors between the treatment arms in both trials. However, the findings regarding the treatment effect in the CPS<5 subgroup were consistent with those of the CheckMate 649 and ORIENT-16. Moreover, different IHC assays (28–8 and Dako 22C3) were used to assess PD-L1 expression in CheckMate 649 and ORIENT-16, which may have led to between-heterogeneity and posed challenges to the pooled analysis. However, good concordance in identifying PD-L1-high and PD-L1-low cases between the 28-8 and Dako 22C3 assays was reported in a previous GC study (Ahn and Kim, 2021), suggesting the potential interchangeability of these two PD-L1 assays (Wang et al., 2022).

In summary, this pooled analysis of two large-scale RCTs in treatment-naïve advanced GC, CheckMate 649 and ORIENT-16, demonstrated that the efficacy of PD-1 blockade plus chemotherapy versus chemotherapy alone significantly attenuated in the CPS<5 subgroup than in the CPS≥5 subgroup. Since the added value of anti-PD-1 therapy to first-line chemotherapy is minimal in patients with a CPS<5, the use of PD-1 inhibitors should be individualized for this patient subset. In addition, major efforts should be made to develop highly efficacious therapeutics beyond the anti-PD-1-chemotherapy combination to improve the survival outcomes of these patients.

## Data Availability

The original contributions presented in the study are included in the article/Supplementary Material, further inquiries can be directed to the corresponding authors.

## Abbreviations

CPS, Combined Positive Score; GC, gastric cancer; HR, hazard ratio; IHC, immunohistochemistry; IPD, individual patient-level data; KM, Kaplan-Meier; OS, overall survival; PD-1, Programmed Cell Death-Protein 1; PD-L1, Programmed Cell Death-Ligand 1; PFS, progression-free survival; RCT, randomized controlled trial; TPS, tumor proportion score.
