# Supplementary material for: PD-L1 expression predicts the efficacy of PD-1 blockade plus chemotherapy versus chemotherapy alone in treatment-naïve advanced or metastatic gastric cancer: a pooled analysis of reconstructed individual patient-level data from two randomized trials
Source: Front Cell Dev Biol. 2025 Jul 3;13:1636288. doi: 10.3389/fcell.2025.1636288 (PMC12267251; doi:10.3389/fcell.2025.1636288)
Supplement: Supplementary file 1 [file DataSheet1.pdf]

Supplementary Materials

Contents:

Figure S1. Literature selection process. 2

Figure S2. Quality assessment of data reconstruction and matching for the chemo-immunotherapy arm in CheckMate 649. 3

Figure S3. Quality assessment of data reconstruction and matching for the chemotherapy-only arm in CheckMate 649. 4

Figure S4. Quality assessment of data reconstruction and matching for the chemo-immunotherapy arm in ORIENT-16. 5

Figure S5. Quality assessment of data reconstruction and matching for the chemotherapy-only arm in ORIENT-16. 6

Table S1 The original reported data and reconstructed data of overall survival and progression-free survival in CheckMate 649 and ORIENT-16. 7

Figure S1. Literature selection process.

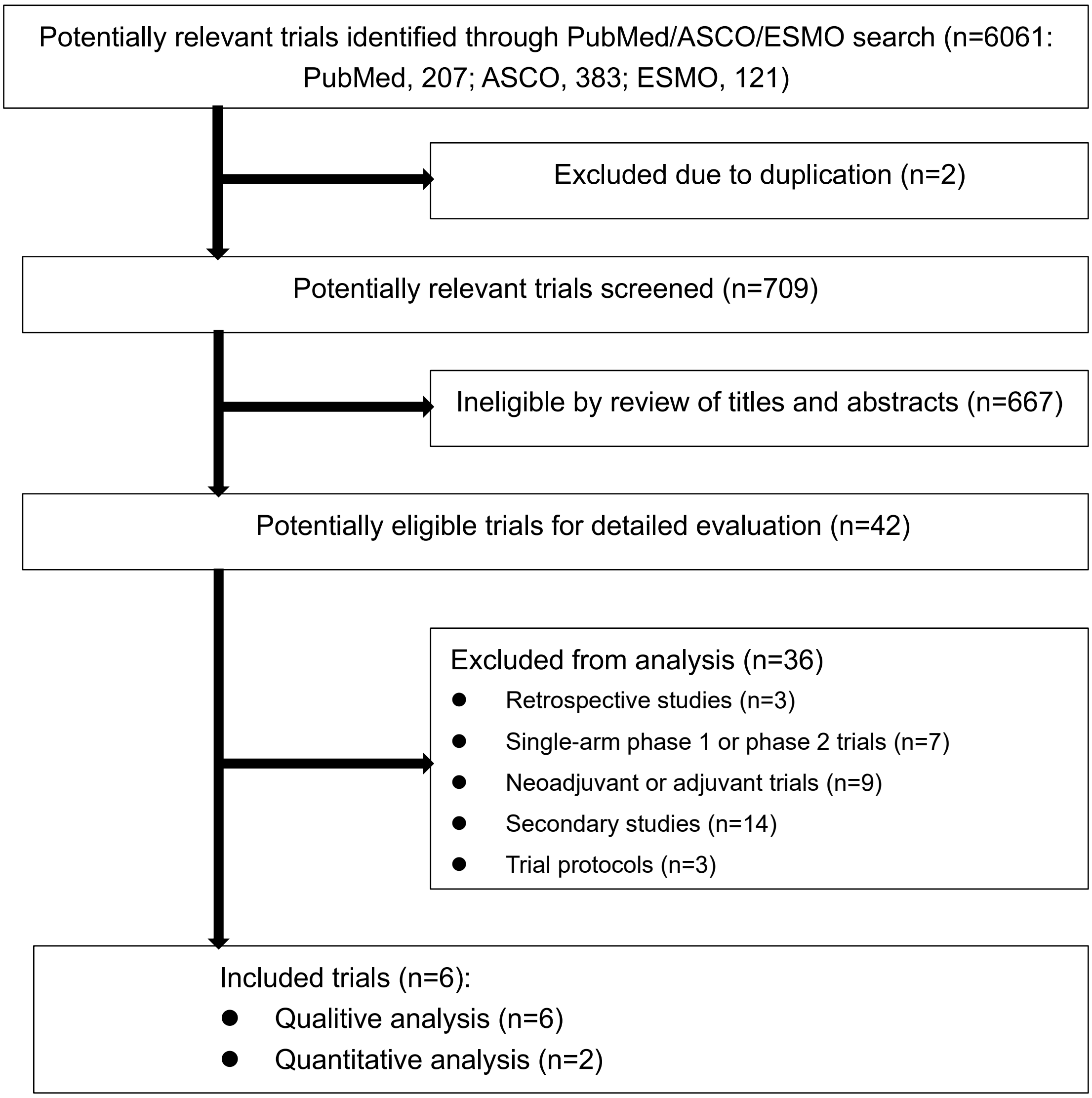

Figure S2. Quality assessment of data reconstruction and matching for the chemo-immunotherapy arm in CheckMate 649.

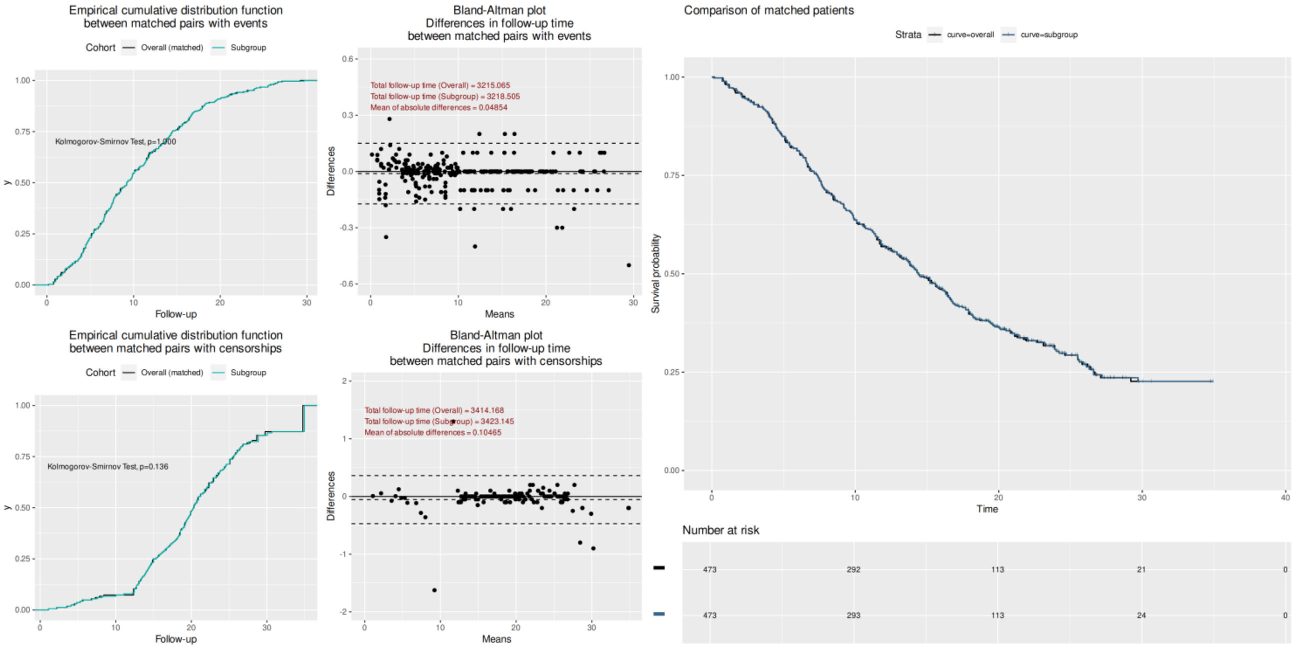

Figure S3. Quality assessment of data reconstruction and matching for the chemotherapy-only arm in CheckMate 649.

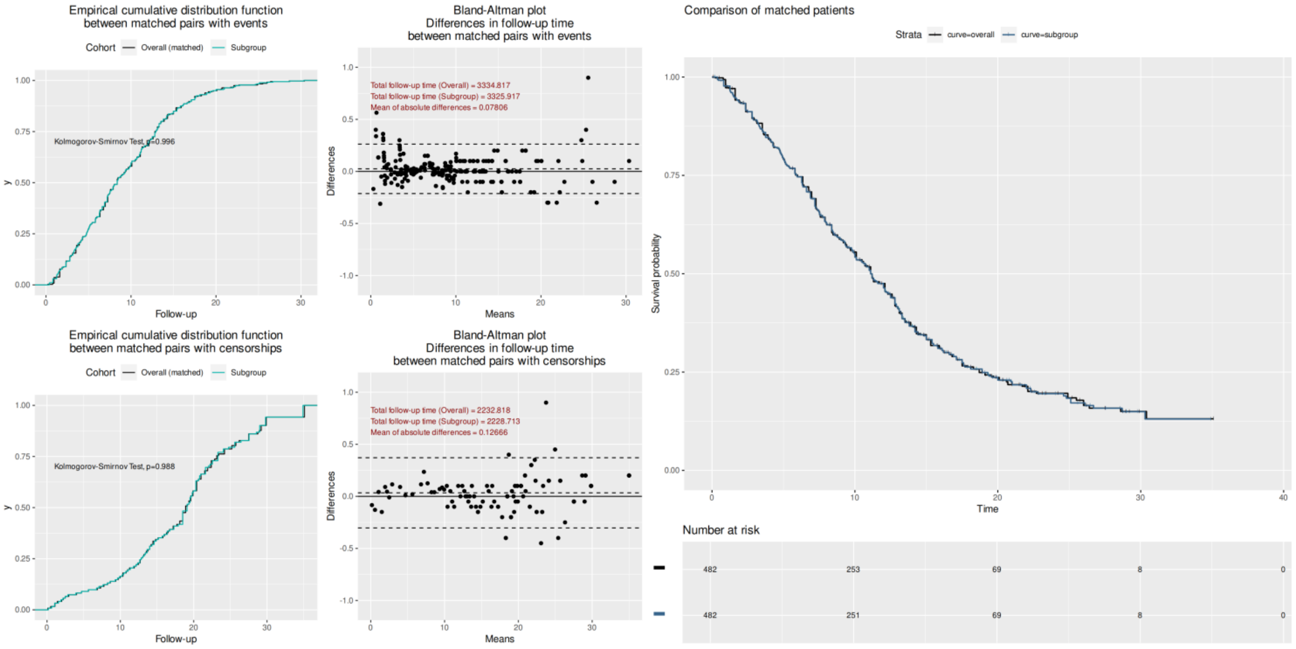

Figure S4. Quality assessment of data reconstruction and matching for the chemo-immunotherapy arm in ORIENT-16.

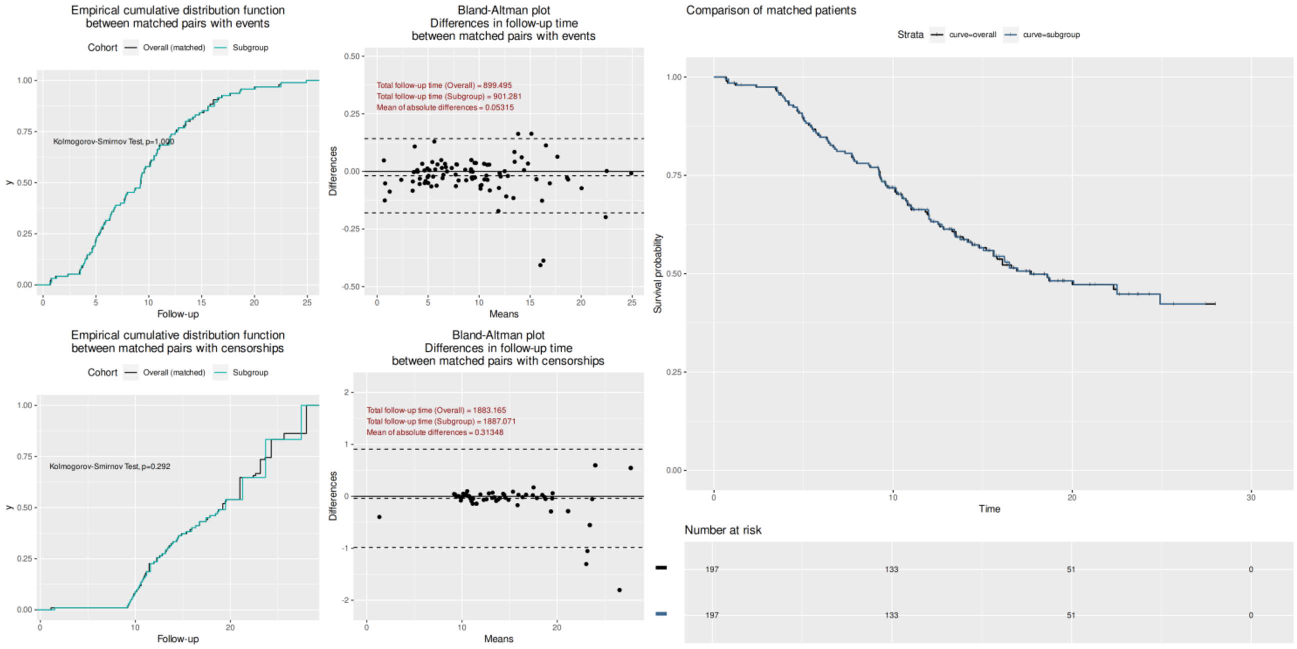

Figure S5. Quality assessment of data reconstruction and matching for the chemotherapy-only arm in ORIENT-16.

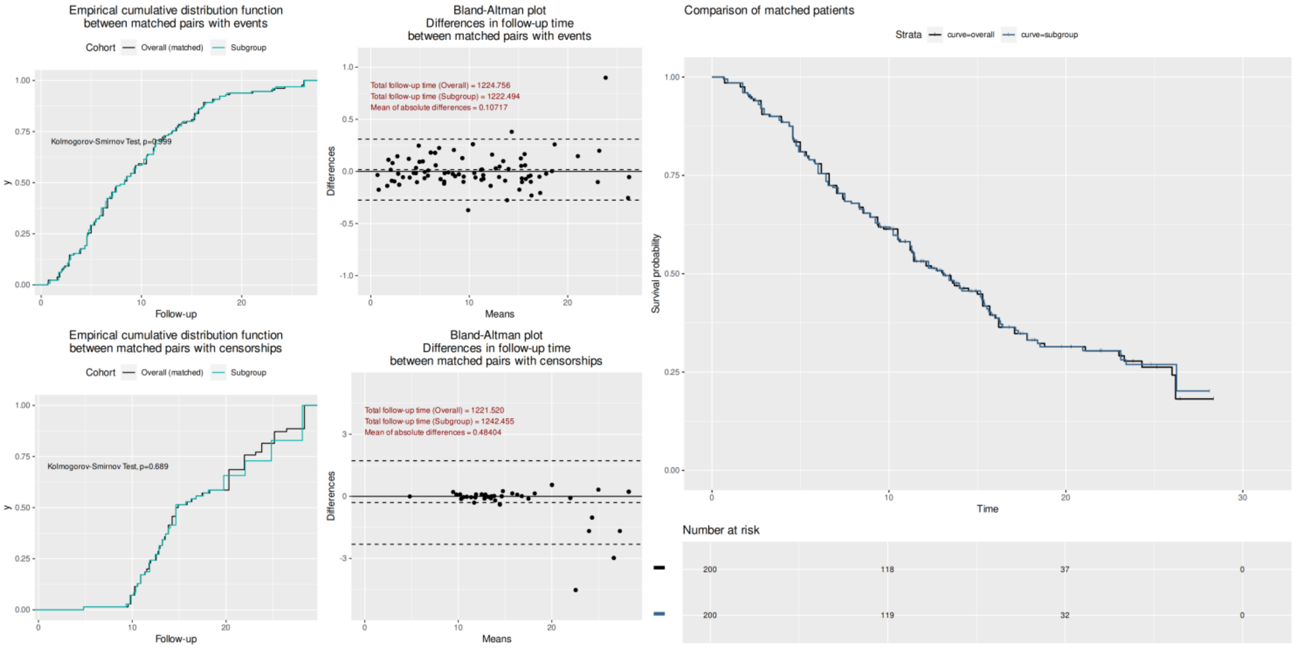

Table S1 The original reported data and reconstructed data of overall survival and progression-free survival in CheckMate 649 and ORIENT-16.

	OS		PFS	
	**Reported**	**Reconstructed**	**Reported**	**Reconstructed**
**HR**				
CheckMate 649, ITT	0.80	0.80	0.77	0.78
CheckMate 649, CPS≥5	0.71	0.71	0.68	0.70
ORIENT-16, ITT	0.77	0.74	0.64	0.67
ORIENT-16, CPS≥5	0.66	0.63	0.63	0.63
**Median, months (Treatment arm)**				
CheckMate 649, ITT	13.8	14.0	7.7	7.7
CheckMate 649, CPS≥5	14.4	14.4	7.7	7.7
ORIENT-16, ITT	15.2	15.6	7.1	7.3
ORIENT-16, CPS≥5	18.4	17.6	7.7	7.3
**Median, months (Control arm)**				
CheckMate 649, ITT	11.6	11.7	6.9	6.9
CheckMate 649, CPS≥5	11.1	11.1	6.0	6.1
ORIENT-16, ITT	12.3	12.6	5.7	6.0
ORIENT-16, CPS≥5	12.9	13.1	5.8	5.8

Abbreviations: CPS: Combined Positive Score; OS: overall survival; PFS: progression-free survival; ITT: Intention-to-treat population.
